# Carbon and Silicon Impurity Defects in GaN: Simulating Single-Photon Emitters by First Principles

**DOI:** 10.3390/ma17153788

**Published:** 2024-08-01

**Authors:** Junxiao Yuan, Jinglei Du, Yidong Hou, Feiliang Chen, Qian Li

**Affiliations:** 1 Department of Physics, Sichuan University, Chengdu 610065, China; yuanjunxiao@stu.scu.edu.cn (J.Y.); dujl@scu.edu.cn (J.D.); houyd@scu.edu.cn (Y.H.); 2 Microsystem and Terahertz Research Center, China Academy of Engineering Physics, Chengdu 610299, China; 3 School of Electronic Science and Engineering, University of Electronic Science and Technology of China, Chengdu 610054, China

**Keywords:** single-photon emitters, atom defect, first-principle calculations, telecommunication band, impurity

## Abstract

Defect single-photon emitters (SPE) in gallium nitride (GaN) have garnered great attentions in recent years due to the advantages they offer, including the ability to operate at room temperature, narrow emission linewidths, and high brightness. Nevertheless, the precise nature of the single-photon emission mechanism remains uncertain due to the multitude of potential defects that can form in GaN. In this work, our systematical investigation with the ab initio calculation indicates that carbon and silicon, as common dopants in gallium nitride, can interact with intrinsic defects in GaN and form new high-speed defect single-photon sources. Our findings identify a ternary defect N_Ga_V_N_C_N_ that possesses a short lifetime of less than 1 ns and a small zero-photon line (ZPL) of 864 nm. In other words, this defect can serve as a high-speed single photon source in the short wavelength window for fiber communication. In sharp contrast, the Si-supported defect N_Ga_V_N_Si_N_ has a higher unoccupied defect energy level which enters the conduction band and is therefore unsuitable for single photon emission. A systematic investigation has been conducted into the potential defects, thermal stability, and single-photon emission properties. The relaxation calculation and self-consistent calculations employed the Perdew–Burke–Ernzerhof exchange-correlation functional and Heyd–Scuseria–Ernzerhof exchange-correlation functional, respectively. These findings indicate the potential for high-performance single-photon sources through carbon or silicon doping of GaN.

## 1. Introduction

The single-photon emitters (SPEs) refer to the light sources that emit only one photon at a time. Their emission properties are typically characterized by low photon number statistics and high single-photon purity [1,2,3,4]. These features make them of significant importance in the fields of quantum information science and quantum communication [5]. For instance, single photons generated by a single-photon source can be employed in the quantum key distribution (QKD) for the purpose of ensuring the security and privacy of quantum-encrypted communications [6]. To realize the single photon emission, it requires one isolated two-level system, wherein the transition of an electron between these two levels is typically accompanied by the emission of one photon [7]. To date, a multitude of single photon sources have been both theoretically and experimentally validated in Group III nitrides, such as gallium nitride (GaN). Defect-based single-photon sources in GaN have attracted significant interest due to their advantageous characteristics, including operation at room temperature, controllable composition, high purity, narrow linewidth, and also the well-established fabrication processes [8,9,10,11,12]. 

The stability of defects in Group III nitrides has an important effect on the properties of the material. Those issues have been researched in previous works [13,14]. Defects in crystal generally include intrinsic defects and impurity defects, which are composed of crystal elements and doping elements, respectively. Recent studies have demonstrated that certain intrinsic point defects and intrinsic defect pairs in GaN are capable of emitting single photons [15,16]. However, the occurrence of SPE resulting from the doping of elements in GaN has not been conclusively demonstrated. Although the majority of single-photon emission results to date have been observed are in unintentionally doped gallium nitride, The potential for impurity defects to form a single photon source cannot be discounted. In 2023, Yifei Geng’ work indicated that the SPE in GaN may be caused by an impurity/vacancy defect pair because the dipole orientations’ angular distributions of such defect pair are in agreement with the experimental results [17].

Carbon is one of the most prevalent unintentional dopants in GaN. The introduction of carbon is almost inevitable during the preparation of GaN thin films using processes such as metal-organic vapor phase epitaxy. For instance, when dimethylgallium and dimethylamine are employed as sources for gallium and nitrogen, respectively, there is a certain probability that carbon atoms from these gases will be incorporated into the fabricated GaN thin film [18,19,20]. The intentional doping of GaN is also a common method to adjust its physicochemical properties in experimental and production settings. For example, silicon, which belongs to the same group as carbon, is one of the most commonly used intentional dopants in GaN [21,22,23]. The incorporation of silicon can significantly increase the electron concentration in GaN, conferring upon it the characteristics of an n-type semiconductor. The incorporation of these newly introduced impurity atoms can result in the formation of defects in the form of substitutions or interstitials. Furthermore, they can combine with intrinsic point defects to form defect pairs [24,25]. Consequently, the existence of these doping atoms can affect the defect levels of GaN single-photon sources, thereby altering their single-photon emission.

In this work, we systematically investigated the influence of carbon and silicon-doped impurity defects on the single-photon emission of GaN through first-principles calculations. Our results identify one ternary defect N_Ga_V_N_C_N_, which exhibits a short lifetime of less than 1 ns, accompanied by a zero-photon line (ZPL) of 864 nm. In other words, this defect can serve as a high-speed single photon source in the short-wavelength window for fiber communication. Compared to N_Ga_V_N_, the radiative lifetime of this ternary defect N_Ga_V_N_C_N_ was reduced by an order of magnitude, indicating a higher emission rate. In sharp contrast, the Si-supported defect N_Ga_V_N_Si_N_ has a higher unoccupied defect energy level which enters the conduction band and is therefore unsuitable for single photon emission. The atomic structures, energy bands, and emission wavelengths of the impurity defects were analyzed through first-principles calculations. 

## 2. Simulation Methods

All of the first-principle calculations in this work were based on the PWmat software package (number version PWMAT 10.0.0.2, lonxun quantum company, Beijing China) [26,27]. The four-atom unit cells of wurtzite gallium nitride with space group P63mc were expanded to a 72-atom supercell consisting of 3 × 3 × 2 primitive cells. After removing or adding atoms to form defects, the supercell was relaxed until the energy error was less than 0.00173 eV. Since structural relaxation calculation required lower calculating accuracy than the self-consistent calculation, the relaxation calculation and self-consistent calculation employed the Perdew–Burke–Ernzerhof (PBE) exchange-correlation functional [28] and the Heyd–Scuseria–Ernzerhof (HSE) exchange-correlation functional [29], respectively. According to the literature [25], this method yields results that are almost indistinguishable in precision from those obtained by using HSE throughout the entire process. All calculations were performed with an energy cutoff of 70 Ry.

## 3. Results and Discussion

### 3.1. Defect Pair Made Up of Impurity and Nitrogen Vacancy

**Figure 1:**
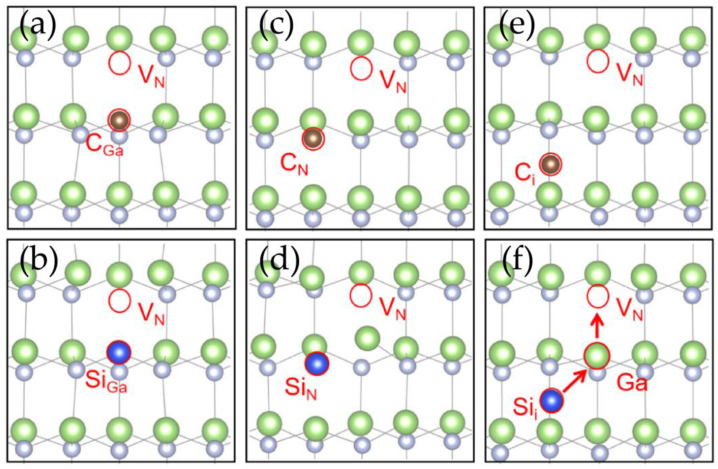
The structure of defect pairs formed by combining impurity atom defects with intrinsic point defects: (**a**) C_Ga_V_N_, (**b**) Si_Ga_V_N_, (**c**) C_N_V_N_, (**d**) Si_N_V_N_, (**e**) C_i_V_N_, and (**f**) Si_i_V_N_. The green, grey, brown and blue represent gallium, nitrogen, carbon and silicon atoms respectively.

For the GaN doped with carbon and silicon, six types of point defects are formed by doping atoms, namely C_N_, Si_N_, C_i_, Si_i_, C_Ga_, and Si_Ga_. For example, C_N_ stands for the defect formed by a nitrogen atom replaced by a carbon atom, and C_i_ stands for an interstitial carbon atom. Recent computational studies have revealed the defect levels and luminescence properties of these six defects [30]. These results indicate that the point defects formed by carbon or silicon do not possess an isolated two-level system that is required for single-photon emission, rendering them incapable of serving as single-photon sources. However, these six defects may potentially combine with the intrinsic point defects in GaN to form defect pairs, which may serve as single-photon sources and have not been investigated in previous works. The nitrogen vacancy defect V_N_ was selected for combination with the doped point defects to form defect pairs. As previously demonstrated in our research [15], V_N_ represents intrinsic defect with the lowest formation energy in GaN. This makes it the defect V_N_ with the highest defect density and the greatest probability of forming a defect pair. In this case, there are six defect pairs that were identified when combining carbon and silicon elements with V_N_, namely, C_Ga_V_N_, Si_Ga_V_N_, C_N_V_N_, Si_N_V_N_, C_i_V_N_, and Si_i_V_N_. Their atomic structures are shown in Figure 1a–e. After structural optimization calculations, most of the defect pairs were found to be capable of stabilizing their structures upon combination with the V_N_ defect, as shown in Figure 1a–e. The C_Ga_V_N_ and Si_Ga_V_N_ formed axisymmetric stable structures in which the axis of symmetry is the axis along the [0001] direction passing through supercell center. C_N_V_N_, Si_N_V_N_, and C_i_V_N_ formed stable structures which have no obvious axis of symmetry or center of symmetry. For the V_N_Si_i_ defect pair, the position of its atoms changes greatly during the structural optimization process, as shown in Figure 1f. The interstitial Si continuously approaches the Ga atom near the V_N_, replacing it to form a Si_Ga_ defect. Meanwhile the displaced Ga atom moves upwards towards the V_N_ defect, combining with it to form a GaN defect. This process results in the gradual forming of a stable Si_Ga_Ga_N_ defect pair.

In the case of V_N_C_i_, defect C_i_ does not approach the Ga atom near the V_N_. This difference between V_N_C_i_ and V_N_Si_i_ can be attributed to the influence of the formation energies of C_i_, C_Ga_, Si_i_, and Si_Ga_. As indicated in Reference [30], the formation energies of C_i_, C_Ga_, Si_i_, and Si_Ga_ are 6.33 eV, 2.63 eV, 11.58 eV, and 2.71 eV, respectively. The formation energy of Si_i_ in GaN is considerably higher than that of Si_Ga_, with an energy difference of up to 8.87 eV. This substantial energy disparity indicates that Si_Ga_ is markedly more stable than Si_i_, thereby facilitating the transformation from Si_i_ to Si_Ga_. Furthermore, the presence of V_N_ results in the absence of a Ga-N bond around Ga atoms, rendering them more susceptible to Si substitution. In contrast, the formation energy of C_i_ is only slightly higher than that of C_Ga_ by 3.70 eV, resulting in a smaller energy gap and thus a weaker tendency for C_i_ to convert into C_Ga_. 

**Figure 2:**
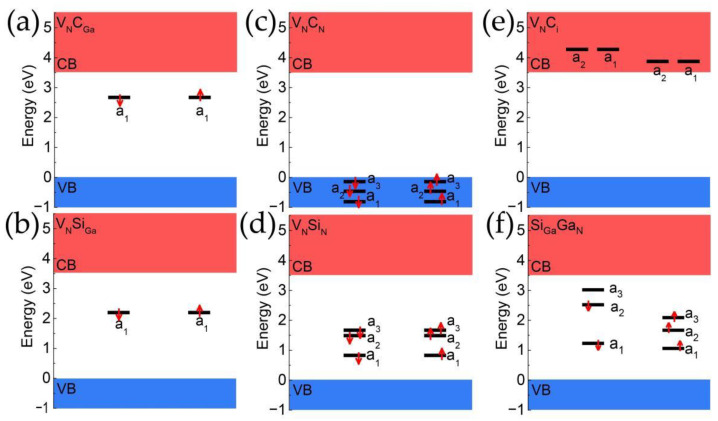
The band structure of defect pairs: (**a**) C_Ga_V_N_, (**b**) Si_Ga_V_N_, (**c**) C_N_V_N_, (**d**) Si_N_V_N_, (**e**) C_i_V_N_, and (**f**) Si_Ga_Ga_N_. The red arrow represents the direction of the electron’s spin.

To assess whether these defect pairs possess the isolated two-level system required for single photon emission, the band structures are systematically investigated, as shown in Figure 2. It can be seen that the defect pairs formed by combining V_N_ with C_Ga_ or Si_Ga_, namely V_N_C_Ga_ and V_N_Si_Ga_, have a similar band structure that features only one defect level, a_1_, within the bandgap. Electrons at this defect level can only transit between the conduction and valence bands and cannot provide single photon emission. The defect pair V_N_C_N_, formed by combining V_N_ and C_N_, have defect levels that are all within the valence band and also cannot emit single photons. For the V_N_Si_N_, all the defect levels (a_1_, a_2_, and a_3_) have been occupied by electrons, so there is no empty level for electron transition. For the Si_Ga_Ga_N_, the a_3_ in spin-down channel is the only empty defect level in band gap, so the electron transition could only happen between a_3_ and a_2_. However, the transition energy between a_2_ and a_3_ is 0.5 eV, which is too small when comparing with the ZPL measured in experiments (about 1.28–2.00 eV in Ref. [8] and 0.9–1.2 eV in Ref. [9]). In summary, none of the intrinsic point defects V_N_ combined with carbon and silicon impurity defects in gallium nitride possess one ideal isolated two-level system and thus cannot serve as a defect single photon source in GaN.

### 3.2. Ternary Defect Made Up of Impurity and N_Ga_V_N_

In addition to the defects formed by two-point defects, three-point defects may also be combined to form new defects [31,32]. Six intrinsic point defects and six impurity defects can form more than one hundred ternary combinations, which is too many to calculate all. To improve the chances of finding an impurity defect SPE, we select an intrinsic defect pair that has been proven to act as a SPE to combine with the impurity defect. Based on our previous research, N_Ga_V_N_ can serve as the source of single photon emission in GaN. This single photon source may combine with carbon or silicon atoms to form novel defect single photon sources. According to the formation energy results from theeh literature [30], carbon substitutional defect C_N_ has the lowest formation energy (2.63 eV in the N rich condition) and are the most prone to occur. Therefore, this work considers the ternary defect pairs N_Ga_V_N_C_N_ and N_Ga_V_N_Si_N_, which are formed by combining the N_Ga_V_N_ defect pairs with C_N_ and Si_N_ impurity substitutional defects, respectively. The impact of binding with impurity defects on the single photon emission properties of N_Ga_V_N_ also is analyzed. 

**Figure 3:**
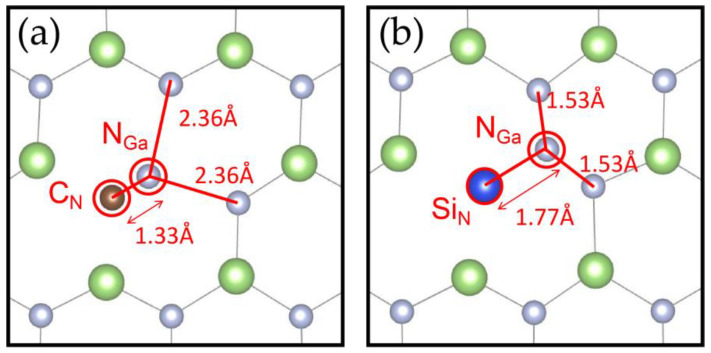
The structure of (**a**) N_Ga_V_N_C_N_ and (**b**) N_Ga_V_N_Si_N_. The green, grey, brown and blue represent gallium, nitrogen, carbon and silicon atoms respectively.

After the optimization of the atomic structure, the configurations of N_Ga_V_N_C_N_ and N_Ga_V_N_Si_N_ are presented in Figure 3. It can be observed that the bond lengths between the substituted nitrogen atom N_Ga_ and C_N_ and S_N_ are 1.33 Å and 1.77 Å, respectively. The C-N bond is 0.44 Å shorter than the Si-N bond. In N_Ga_V_N_C_N_, the substituted nitrogen atom N_Ga_ is observed to move in close proximity to the carbon atom when compared to its initial position before structural optimization. In contrast, in N_Ga_V_N_Si_N_, the substituted nitrogen atom N_Ga_ is observed to move away from the silicon atom. This phenomenon may be attributed to the fact that the Si atom possessed one additional electron shell compared to the N atom, which results in a larger atomic radius and a more pronounced repulsive force that propels the substituted nitrogen atom N_Ga_ away from the center. Conversely, the C atom has one fewer electron than that of the N atom and thus exhibits weaker repulsion, which attracts the substituted nitrogen atom N_Ga_ towards the carbon atom.

**Figure 4:**
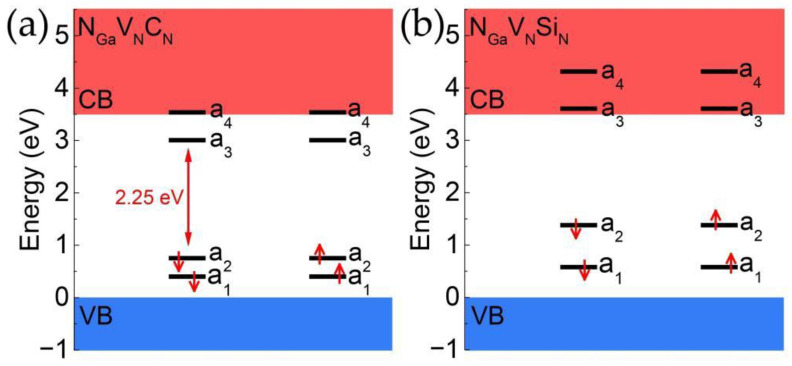
The band structure of (**a**) N_Ga_V_N_C_N_ and (**b**) N_Ga_V_N_Si_N_. The red arrow represents the direction of the electron’s spin.

The calculated band structure diagrams of the N_Ga_V_N_C_N_ and N_Ga_V_N_Si_N_ defects are shown in Figure 4. Although the defect level of N_Ga_V_N_C_N_ and N_Ga_V_N_Si_N_ are similar to each other, the a_3_ levels of the N_Ga_V_N_C_N_ defect is within the bandgap and detached from the conduction band. For N_Ga_V_N_C_N_, the a_1_ and a_2_ were occupied by electrons and the a3 was the only empty defect level in band gap. This allows it to form an isolated two-level system with the a_2_ and thereby enables it to serve as an SPE. For N_Ga_V_N_Si_N_, the a_1_ and a_2_ were occupied by electrons and there is no empty defect level in band gap. The a_3_ energy level of the N_Ga_V_N_Si_N_ is within the conduction band, which precludes the formation of an isolated two-level system and thus incapable of functioning as an SPE.

To analyze the stability of ternary defects, the formation energy of N_Ga_V_N_C_N_ was calculated according to Equation (1)

*E*_f_(α,q,*E*_F_) = *E*_tot_(α,q) − *E*_tot_(host) + ∑*n*_i_*μ*_j_+ q(*E*_F_ + *E*_VBM_(host)) + *E*_corr_ (1)

where E_tot_(α,q) is the total energy of the defect type α with charge q. E_tot_(host) is the total energy of a perfect supercell without any defect. E_corr_ is d a corrected value for the long-range image-charge Coulomb interaction and E_VBM_(host) is the energy corresponding to the valence band maximum, respectively. The alignment of E_VBM_(host) is based on the electrostatic potential farthest from the center of the supercell [33]. μi is the chemical potential where i is the element type. n_i_ is the number of atoms i that are removed from the cell. E_F_ is the Fermi level which depends on the electron concentration. For doped semiconductors, a high Fermi level typically corresponds to a high electron concentration, i.e., an n-type environment.

**Figure 5:**
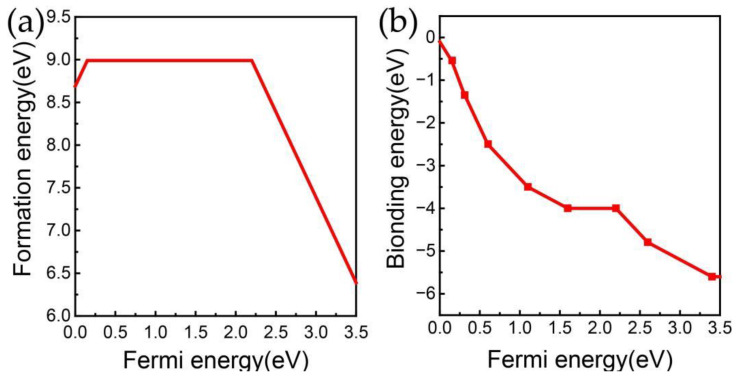
The thermal stability of N_Ga_V_N_C_N_; (**a**) formation energy, (**b**) binding energy.

For defect N_Ga_V_N_C_N_, the GaN supercell loses one nitrogen atom and one gallium atom, making the number of remaining nitrogen atoms equal to the number of gallium atoms, so its chemical potential is independent of the nitrogen-rich or gallium-rich environment. The extra carbon atom is not an intrinsic defect of gallium nitride and is also not affected by nitrogen-rich or gallium-rich environments. The formation energy of N_Ga_V_N_C_N_ under both nitrogen-rich and gallium-rich conditions is represented by the same curve, as shown in Figure 5a. The neutral charge state of N_Ga_V_N_C_N_ corresponds to a line segment with slope of 0 in the formation energy curve, which is a broad energy region (0.2–2.2 eV Fermi energy). This suggests that the neutral charge state can exist in p-doped, n-doped, and unintentionally doped environments.

The binding energy of N_Ga_V_N_C_N_ was calculated according to Equation (2)

*E*_b_(N_Ga_V_N_C_N_,q_1_,E_F_) = *E*_f_(N_Ga_V_N_C_N_,q_1_,E_F_) − *E*_f_(N_Ga_V_N_,q_2_,E_F_) − *E*_f_(C_N_,q_3_,E_F_) + (q_1_ − q_2_ − q_3_)*E*_F_ (2)

where E_f_(N_Ga_V_N_C_N_), E_f_(N_Ga_V_N_), and E_f_(C_N_) are the formation energies of N_Ga_V_N_C_N_, N_Ga_V_N_, and C_N_, respectively. As shown in Figure 5b, the binding energy decreases with increasing the Fermi level and are always less than zero. This implies that once the N_Ga_V_N_C_N_ defect is formed, it requires additional energy absorption for separation.

**Figure 6:**
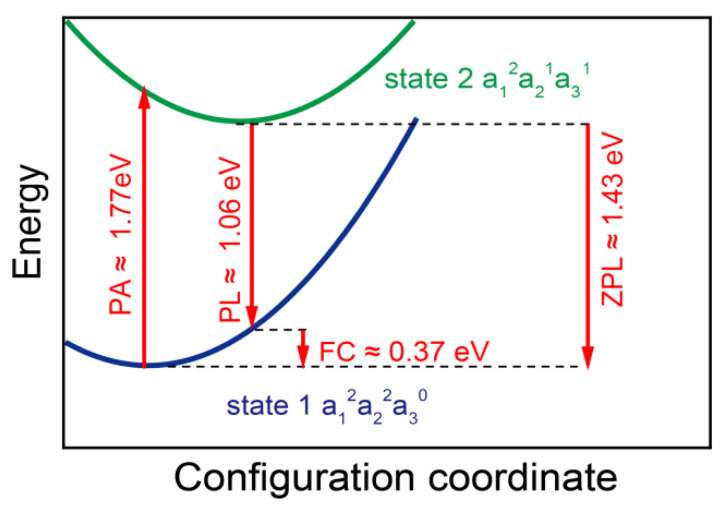
The configuration coordinate diagram of N_Ga_V_N_C_N_.

To analyze the single-photon emission performance of N_Ga_V_N_C_N_, we calculated its zero-phonon line(ZPL) and luminescence lifetime. Figure 6 depicts the configuration coordinate diagram of the optical transition in N_Ga_V_N_C_N_. State 1 represents the ground state which has an electron configuration of a_1_^2^a_2_^2^a_3_^1^, and state 2 is the excited state formed by exciting an electron from a_2_ to a_3_. The photo absorption (PA) process involves an electron transition from the ground state to the excited state with the same atom configuration. The photoluminescence (PL) process is an electron from the excited state to ground state with the same atom configuration. The ZPL is the energy difference between the lowest energy of states 1 and 2, which represents an electron transition process without the influence of phonon. The FC (Franck–Condon) of 0.37 eV indicates a redshift of the PL peak with respect to the ZPL peak. The calculated PA energy of 2.15 eV suggests that the SPE can be excited by a visible laser, such as the commercial 700 nm laser. The calculated PA energy of 2.15 eV suggests that the SPE can be excited by a visible laser, such as the commercial 700 nm laser. The ZPL wavelength of N_Ga_V_N_C_N_, which is about 864 nm, experiences a noticeable redshift when compared with that of N_Ga_V_N_ (626 nm in Ref. [15]). This wavelength is situated within an important window in the communication band, specifically the short-wavelength window (850 nm), which is conducive to serve as the multimode fibers [34].

The radiative lifetime τ associated with ZPL could judge the SPE quality, which is given as 1/r_ij_. The transition rate r_ij_ can be calculated by Fermi’s golden rule as:

(3) $$r_{\mathrm{ij}\;}=\frac{\omega^{3}n{\left|\mu_{\mathrm{ij}}\right|}^{2}}{3\pi \varepsilon_{0}\text{ℏ}c^{3}}$$

where ħ is the reduced Planck constant, ω is the frequency of the emitted photon of ZPL, |μ_ij_| is the transition dipole moment from state i to state j, n is the refraction index, ε_0_ is the vacuum permittivity, and c is the speed of light in vacuum. The calculated lifetimes were shown in Table 1, which compares the single-photon emission performance parameters of N_Ga_V_N_C_N_ with N_Ga_V_N_. The N_Ga_V_N_ defect pair exhibits a remarkably short lifetime of 0.31 ns, which is approximately one-tenth that of N_Ga_V_N_. This indicates that N_Ga_V_N_C_N_ has a higher single-photon emission rate than N_Ga_V_N_. This is of significant value for meeting the demands of high-speed and high-brightness emission in the field of quantum information. Another example is the field of random number generation, where a short-lived single-photon source can be used to generate larger-scale quantum random numbers. The larger the scale of random numbers, the more complex the encryption provided in cryptography.

## 4. Conclusions

This study calculates the impact of carbon and silicon, two common doping environments, on single photon emission in gallium nitride. Initially, six defect pairs were selected, formed by combining native point defects V_N_ with non-native defects corresponding to carbon and silicon, with the exception that the defect pair V_N_Si_i_ is unstable. These defect pairs exhibited band structures indicating that they cannot form isolated two-level systems within the band gap necessary for single photon emission. Consequently, they cannot serve as single photon sources. Subsequently, we calculated the ternary defects N_Ga_V_N_C_N_ and N_Ga_V_N_Si_N_ formed by combining the single photon source N_Ga_V_N_ with the most easily formed non-native point defects C_N_ and Si_N_. Our results identified one ternary defect N_Ga_V_N_C_N_ which exhibited a short lifetime of less than 1 ns and a small zero-photon line (ZPL) of 864 nm. In other words, this defect can serve as a high-speed single photon source in the short-wavelength window for fiber communication. In sharp contrast, the Si-supported defects N_Ga_V_N_Si_N_ has a higher unoccupied defect energy level which enters the conduction band and is not suitable for single photon emission. The findings of this work provide guidance for altering the performance of single photon emission in gallium nitride through carbon and silicon doping.

In this study, we calculate and analyze only those defects that are most likely to act as single-photon sources. Nevertheless, in the environment of carbon and silicon doping, there are still more than a hundred distinct defect pairs and ternary defects that require extensive computational analysis. The integration of artificial intelligence with first-principles calculations may facilitate the optimization of computational efficiency.

## Figures and Tables

**Table 1:** Zero-phonon line, transition rate, and luminescence lifetime of N_Ga_V_N_C_N_ and N_Ga_V_N_.

Defect	ZPL	Lifetime	Emission Rate (per Second)
N_Ga_V_N_	1.98 eV (626 nm)	3.56 ns	2.8 × 10^8^
N_Ga_V_N_C_N_	1.43 eV (864 nm)	0.301 ns	3.3 × 10^9^

## Data Availability

Data are contained within the article.
